# Moderate Wine Consumption and Gastrointestinal Diseases

**DOI:** 10.3390/nu17101608

**Published:** 2025-05-08

**Authors:** Patrizia Restani, Chiara Di Lorenzo, Arina Oana Antoce, Marcos Araujo, Corinne Bani, Francesca Mercogliano, Jean-Claude Ruf, Rena I. Kosti, Pierre-Louis Teissedre

**Affiliations:** 1 Faculty of Pharmacy, Università degli Studi di Milano, 20133 Milano, Italy; patrizia.restani@unimi.it; 2 CRC “Innovation for Well-Being and Environment”, Università degli Studi di Milano, 20122 Milano, Italy; 3 Department of Pharmacological and Biomolecular Sciences, Università degli Studi di Milano, 20133 Milano, Italy; corinne.bani@unimi.it (C.B.); francesca.mercogliano@unimi.it (F.M.); 4 Department of Bioengineering of Horti-Viticultural Systems, University of Agronomic Sciences and Veterinary Medicine of Bucharest, 011464 Bucharest, Romania; arina.antoce@usamv.ro; 5 OIV—International Organisation of Vine and Wine, 21000 Dijon, France; sanco@oiv.int (M.A.); cst@oiv.int (J.-C.R.); 6 Department of Nutrition and Dietetics, School of Physical Education, Sports and Dietetics, University of Thessaly, 42132 Trikala, Greece; renakosti@uth.gr; 7 Université de Bordeaux, Bordeaux INP, INRAE, OENO, UMR 1366, ISVV, F-33140 Villenave d’Ornon, France; pierre-louis.teissedre@u-bordeaux.fr

**Keywords:** gastrointestinal diseases, wine, alcoholic beverages, inflammatory bowel disease or IBD, irritable bowel syndrome or IBS, gastroesophageal disease or GERD

## Abstract

By conducting a narrative review of the scientific literature, the authors of this study sought to verify whether there were sufficient data to answer the following question: “Can wine positively or negatively influence the incidence and severity of disorders associated with gastrointestinal (GI) diseases?”. In this review, most of the studies considered tested different alcoholic beverages (other than wine), not always reporting in the conclusions the possible difference in the extent of symptoms. Although alcohol certainly plays a central role in influencing the oesophageal and gastric environment, no studies evaluating the role of alcohol as such were included, since the aim of the review was to understand whether wine can be moderately consumed by patients with gastrointestinal diseases. The analysis of studies selected from the main reference databases indicates that even moderate wine consumption can be a source of discomfort in subjects with the GI diseases included in this review (gastritis and gastroesophageal disease, gastrointestinal motility, inflammatory bowel disease, irritable bowel syndrome, and microscopic colitis). This does not mean that a certain percentage of patients cannot tolerate moderate amounts of alcoholic beverages; however, discussion with the family doctor or specialist is essential to identify the correct diet in which to include or exclude the consumption of wine. One of the limitations of this review is the low number of studies available, at least for some of the pathologies considered. It is important to emphasise, however, that some selected epidemiological studies, which include many subjects (even over 100,000), can provide useful information from a scientific point of view.

## 1. Introduction

Gastrointestinal disorders (GIDs) are among the most common and widespread clinical problems worldwide. The study conducted by Sperber et al. (2021) involving 73,076 adults from 33 countries and six continents showed that over 40% of the interviewed participants suffered from GIDs, which required the use of healthcare and affected their quality of life [1].

Diet plays a well-defined role in the induction, severity of symptoms, and progression of many gastrointestinal (GI) diseases, although the identification of the role of nutrient classes or single molecule on the different disorders is quite complex. In fact, many confounding factors, such as smoking or physical activity, can invalidate the scientific robustness of the data that result from epidemiological or clinical studies. A possible link between the “Western diet” and the worsening of symptoms in GIDs seems to be confirmed, and the data indicate that a controlled intake of animal fats and simple sugars is necessary in all patients affected by any GIDs [2,3]. The World Health Organization (WHO) takes extremely restrictive positions towards all alcoholic beverages and suggests that there is no dose of alcohol without risk [4]. Despite this, the moderate consumption of wine has been associated with positive properties, e.g., for the cardiovascular [5] and nervous systems [6]. Since wine is traditionally included in the Mediterranean diet, which is considered healthy, the aim of this study was to verify whether wine may positively or negatively influence the incidence and severity of disorders associated with GI diseases, particularly related to stomach and intestinal disorders. The available data are critically evaluated with the awareness that wine itself could play a complex role in GI diseases, acting both as a risk factor for their development and as a contributor to exacerbating symptoms in existing conditions.

Some promising results have been obtained by setting exclusion diets, such as the well-known low-FODMAP approach [7,8]. Further information is included in the sections of this review dedicated to selected diseases.

### Grapes, Juices, and Alcoholic Beverages in GI Diseases

Grapes and their derivatives are not banned from the diets of subjects with gastrointestinal problems. In the case of the FODMAPs diet, mentioned above, fruit or vegetable juices from some foods (banana, strawberries, blueberries, citrus fruits, melon, kiwi, papaya, passion fruit, and grapes) and wine in moderate quantities are allowed [7]. More specific indications are given in the description of the different gastrointestinal pathologies. This is not to deny that alcohol represents a risk for the development of gastrointestinal diseases [9], and permissible wine consumption always refers to moderate use with meals (even if the term “moderate” is far from an internationally agreed definition).

## 2. Materials and Methods

The most important scientific databases of references and abstracts in the life sciences (PubMed, MEDLINE, Embase, CAB-Abstract) were systematically searched (from database inception to February 2025) using the terms “grape” or “wine” or “*Vitis vinifera*”, in combination with “gastrointestinal disease” or “gastrointestinal disorders”, as well as celiac disease, Crohn’s disease, constipation, oesophageal reflux, gastritis, inflammatory bowel disease (or IBD), intestinal mobility, irritable bowel syndrome (or IBS), and ulcerative colitis. We refined the results for "human studies" and "controlled trials". This review is focused on stomach and intestinal disorders, while the effects of wine on the liver and pancreas were not included and will be considered in specific future literature reviews. Studies focusing on the risk or progression of cancer were excluded, since they have been considered previously [10].

Reviews and studies including alcohol without any distinction in terms of the alcoholic beverage type were excluded. Moreover, studies performed in vitro, in laboratory animals, or regarding purified molecules were considered only when they could be used to improve the discussion or to suggest mechanisms responsible for the effect observed in human studies. The studies were then selected according to their compliance with the chosen inclusion criteria.

A separate literature search was performed using the terms “grape” or “wine” or “*Vitis vinifera*”, in combination with “microbiota”, to include some observations on this topic, which is closely related to the health of the intestinal tract. As addressed below, the microbiota is mentioned only if it was considered and discussed in the clinical studies collected for this review.

Although the literature search followed a structured and methodical approach, the present work is conceived as a narrative review aimed at providing a critical and comprehensive synthesis of the available evidence rather than a formal systematic assessment.

## 3. Results and Discussion

This review considers those pathologies affecting the gastrointestinal system for which scientific publications dealing with the relationship between the pathology and wine consumption have been found. Each pathology is introduced with a short description to make the text more understandable to all readers. For the definition of the pathologies considered, reference is made to the same bibliographic sources [11,12,13,14]. The studies that met the chosen inclusion criteria are summarized in Table 1.

### 3.1. Celiac Disease

Celiac disease (CD) was included in a previous review when the role of moderate wine consumption in immune diseases was considered [41]. It is treated here only briefly. CD is a chronic autoimmune disease associated with gluten, a protein complex present in some cereals (mainly wheat, barley, and rye). Among the characteristics of the pathology, there is the inflammatory state of the small intestine with consequent malabsorption. No study was identified in the literature (using the inclusion criteria of this review) that highlights the role of wine in the positive or negative modulation of intestinal problems in celiac consumers. However, it is important to emphasize that intestinal problems are significant only before the diagnosis of CD but, following the introduction of the gluten-free diet, small intestine problems are greatly attenuated or even overcome [42,43]. Therefore, as healthy consumers, celiac subjects, when stabilized, can drink moderately alcoholic beverages (wine, vodka, gin, whiskey, brandy, rum, etc.) [44].; the only risk would come from the clarification of wine with agents containing gluten, an oenological practice previously used [45], which has recently been abandoned [46].

### 3.2. Gastritis and Gastroesophageal Disease

Gastritis groups together a series of conditions that have in common the inflammation of the stomach lining. There are several recognized causes: stress, infection by *Helicobacter pylori*, the chronic use of nonsteroidal anti-inflammatory drugs (aspirin, ibuprofen, etc.), the abuse of some foods (such as spices and caffeine-containing beverages), and alcohol abuse. Gastritis can appear in acute form following specific stress or develop over time (chronic gastritis). Although it can evolve into an ulcer and can lead to an increased risk of stomach cancer, gastritis usually has a benign evolution and improves quickly with pharmacological treatment (for further details, see the review by Rugge et al. 2021 [47]).

Rare human studies consider the association between wine consumption and gastritis; only that conducted by Gao et al. (2009) met the established selection criteria [15].

The authors evaluated the association between alcohol consumption (e.g., wine or beer) and the presence of chronic atrophic gastritis (CAG) in 9444 elderly subjects recruited in Germany. The quantification of serum pepsinogens I and II was performed for the definition of CAG; a *Helicobacter pylori* antibody assay was required to assess the presence of a current or past infection, which is a key risk factor for this disease. Moderate alcohol consumption (classified as current for <60 g/week or lifetime for ≤51,376 g) was found to be associated with a significantly lower risk of CAG compared with that of abstainers (adjusted odds ratio of 0.71). This inverse association was evident in both moderate beer and wine consumers. The results support the hypothesis that the effect of moderate alcohol consumption may be due at least in part to the facilitation of *H. pylori* clearance.

Gastroesophageal reflux is a condition in which stomach acid repeatedly flows back up into the esophagus. Often referred to as GERD (Gastroesophageal Reflux Disease), this condition, when prolonged, causes irritation of the lining of the esophagus. Most people can manage the discomfort of GERD with lifestyle changes, antacids, and other medications, but some cases of chronic disease can evolve to malignancy and/or require more serious interventions such as surgery. For further details on GERD, refer to the review conducted by Maret-Ouda et al. (2020) [48].

Although alcohol consumption is commonly associated with an increased risk of GERD [49], data found in the scientific literature remain contradictory [50]. Considering the limited number of available studies, this uncertainty becomes even more significant when a single alcoholic beverage is taken into consideration. Of the four studies selected based on the inclusion criteria of this review, only one was performed on subjects with GERD [19]; in the other three cases, the results were obtained from healthy volunteers [16,17,18]. An increase in reflux was observed in both healthy and GERD participants. In their conclusions, Grande et al. (1997) recommended that patients with GERD avoid alcoholic beverages, while Pehl et al. (2006) suggested minimizing the intake of wine or alcoholic beverages, at least in the absence of therapy with gastric acid regulators [16,19].

### 3.3. Gastrointestinal Motility

The term “gastrointestinal motility” refers to the movements of the digestive system that allow the correct transit of the contents within it. When the digestive tract, for various reasons, does not function with normal strength and coordination, symptoms may appear. Each part of the GI tract (esophagus, stomach, small intestine, and large intestine) has a unique function to perform in the digestion process and each has its own distinct motility action. If the coordination of these functions is not harmonious, abnormalities in motility may appear; they may represent symptoms of various pathologies [51].

Dyspepsia, commonly known as “slow digestion”, is an organic or functional disorder of the stomach that usually occurs after meals; it is characterized by a feeling of fullness, abdominal swelling and heartburn. Dyspepsia is widespread in the general population, showing a prevalence ranging from 20 to 40% [52].

Regarding the effect of alcoholic beverages on gastric emptying, the data collected from the selected studies lead to conflicting conclusions. According to some studies selected in this review, wine and other alcoholic beverages, such as beer, inhibit gastric emptying after a solid meal [20,21]. Although alcohol certainly plays a role in the described effect [20,23], wine and beer showed a greater inhibitory action than the corresponding alcoholic solutions [20,21]; this would suggest a role for molecules other than alcohol in inhibitory action. The relatively small influence of alcohol on gastric emptying was confirmed by Moore and colleagues (1981), in a study in which the administration with a meal of a Cabernet wine as such (a 9.5% alcohol) or with a significant reduction in alcohol content (1.13%) induced the same effect on gastric emptying [24]. No difference was observed when the subjects followed the same protocol without a meal.

The possible inhibitory effect on gastric emptying is undesirable in subjects who already suffer from dyspepsia, as the associated symptoms would be accentuated. The use of wine should therefore be carefully considered in subjects who have severe forms of this disorder [52].

In contrast to dyspepsia, there is the so-called dumping syndrome, a condition in which ingested food enters the intestine too quickly, preventing the stomach from carrying out its functions. Dumping syndrome is a frequent complication of gastric and esophageal surgery, such as vagotomy [53]; it is characterized by symptoms such as nausea and vomiting, diarrhea, severe abdominal pain due to the distension of the small intestine, palpitations, and confusion.

Gonzales and colleagues (2020) suggested moderate wine intake as a possible “therapeutic” approach in patients suffering from the dumping syndrome [22]. The authors hypothesized that the anticholinergic effect of wine could lead to a further slowing of gastric emptying in these patients, with the consequent improvement of symptoms.

Constipation is the condition most frequently associated with gastrointestinal transit abnormalities, especially in the elderly and in women. Constipation is defined as having fewer than three bowel movements per week or difficulty in passing stools. It is a fairly common problem, often caused by reduced intake of dietary fiber, fluids, and a lack of physical exercise (primary constipation). Other medical conditions or certain medications may be the cause (secondary constipation). The overall prevalence of constipation varies significantly depending on the definition used and the population included, so that it can range between 2 and 27% of the general population [54].

Constipation is usually treated with qualitative or quantitative changes in the diet, increased physical activity, and, in some cases, with the administration of over-the-counter products. Long-term constipation, also called chronic constipation, may require treatment if it is due to an associated pathology. For further details, see the review by Bharucha and Lacy (2020) [55].

Alcoholic beverages are not commonly associated with constipation problems, according to the list of causes published on the website of the National Institute of Diabetes and Digestive and Kidney Diseases (2018) [56]. However, there are factors associated with alcohol consumption that could worsen the situation in people who already suffer from constipation. These include the following:

- Dehydration. Alcohol increases diuresis, with the greater excretion of liquids. In the event of dehydration, the colon absorbs more water, making the stool harder and more difficult to expel [57].
- Gastrointestinal irritation and inflammation: these conditions can be involved in promoting constipation [58].
- Alcohol can influence the intestinal microbiota, worsening constipation [59].

Contrary to what is reported above for alcohol, the study conducted by Müller-Lissner et al. (2005) indicates that a moderate consumption of beer and wine can make stools softer [25]. The effect of wine was observed in both healthy subjects (30%) and in subjects with constipation (21%) or irritable bowel syndrome with constipation (8%).

### 3.4. Inflammatory Bowel Diseases (IBD)

IBD is a chronic disease, the main symptoms of which are irritation and ulcers in the gastrointestinal tract; the most frequently diagnosed forms are Crohn’s disease (CRD) and ulcerative colitis (UC). The onset of IBD can occur at any age but it usually appears between the ages of 15 and 30. For some patients, IBD is only a mild disease; in other cases, it is a debilitating condition that can lead to the impairment of vital functions with serious consequences.

#### 3.4.1. Crohn’s Disease

Crohn’s disease (CRD), also called ileitis or enteritis, is an inflammatory disease of the gastrointestinal (GI) tract; the affected area can be localized from the mouth to the anus, but it most commonly involves the ileum. The disease presents with swelling that extends deep into the lining of the affected organ, causing pain and the frequent emptying of the bowels (diarrhea). Because the symptoms of Crohn’s disease are like those of other diseases (irritable bowel syndrome and ulcerative colitis), the diagnosis can be difficult. Crohn’s disease affects men and women equally and shows a genetic predisposition; it is estimated that about 20 percent of people with Crohn’s disease have a blood relative with some form of inflammatory bowel disease. Most people are diagnosed with Crohn’s disease in their 20s and 30s. For further details about CRD, see Pasternak et al. (2023) [60]. Many patients with CRD complain of abdominal discomfort after alcohol intake. 

#### 3.4.2. Ulcerative Colitis

Ulcerative colitis (UC) causes inflammation and sores, called ulcers, in the lining of the rectum and colon. Ulcers result from the loss of cells that commonly line the colon. If localized in the rectum and lower colon, it is called ulcerative proctitis. Diagnosing ulcerative colitis is sometimes difficult because the symptoms are very similar to those of other intestinal diseases and Crohn’s disease. Ulcerative colitis is diagnosed equally in males and females and usually appears between the ages of 15 and 30. It appears to have a hereditary component as it is more common in people who have already had other cases diagnosed in family members (20%). For further details on UC, see Gros and Kaplan (2023) [61].

Based on the selected studies, it is not easy to draw particular conclusions regarding the role of wine consumption on the incidence of IBD or on the recurrence of symptoms in subjects who have already been diagnosed. Several critical issues are responsible for this uncertainty: (1) most authors conflate the two forms of IBD (Crohn’s disease and ulcerative colitis) without discussing the possible differences; (2) in some cases, the type of alcoholic beverage or the quantity consumed by patients is not precisely defined; (3) in some studies, the description of the medical history or the study protocol is not satisfactory; (4) the parameters considered and the methodologies used by the authors to define the severity of clinical symptoms may be significantly different and therefore make comparisons difficult. In summary, the relationship between diet and factors involved in the development and/or treatment of the disease is still controversial and not yet fully clarified. The available clinical data indicate that the dietary approach is critical in the remission phase of IBD, although guidelines on optimal diets are still lacking [62]. Among the various concerns regarding the diet of a subject with IBD, there is concern about the consumption of alcoholic beverages, which is generally discouraged to avoid a worsening of symptoms.

In more detail:

1. Studies showing wine consumption to have neither a positive nor a negative role:

In the prospective cohort study performed by Casey and co-workers, lasting 30 years and involving 237,835 participants, no significant association was observed between wine intake and the risk of IBD (both CRD and UC) [26]. Similar results were obtained in a study in which alcohol consumption was compared in 167 patients with a diagnosis of UC and 167 control subjects [30].

2. Studies that suggest the positive role of wine consumption:

In an intervention study performed in 10 UC patients, the moderate consumption of red wine (125 mL × 2/day) was associated with an improved serum iron concentration (anemia biomarker) and the reduced severity of gastrointestinal symptoms [33,34]. The prospective cohort study conducted by Liu and co-workers suggests that, when compared to abstainers, the patients consuming red wine showed a 12–16% reduction in the risk of developing IBD [28].

3. Studies that suggest the negative role of wine consumption:

In the same epidemiological study cited above, Liu and colleagues suggested that subjects who frequently consumed high doses of white wine or champagne may have a 12% increased risk of developing inflammatory bowel disease compared to abstainers [28]. Magee and co-workers suggest a rather unexpected correlation between the sulfite content of alcoholic beverages and the worsening of gastrointestinal symptoms in subjects with UC. The authors based this conclusion on the observation that the negative effects on GI symptoms were attributable to wine and beer but not to liquors that do not contain sulfites. Therefore, they exclude or minimize the role of alcohol in the painful symptoms of UC [29]. The role of sulfur and sulfates, which are contained in some alcoholic beverages, was also suggested by Jowett and co-workers (2004) [63].

Of the 90 subjects with IBD included in the cross-sectional study performed by Swanson et al., 56 consumed alcoholic beverages. Of these, 75% of the subjects reported experiencing a worsening of gastrointestinal symptoms; however, this effect did not depend on the type of beverage or the amount of alcohol consumed [31]. In a cross-over intervention study, Hey and colleagues observed that, compared to controls, subjects with CRD showed an increase in abdominal pain when consuming alcoholic beverages [27]. On the other hand, there was a difference in the severity of symptoms that was lower in the case of wine (red and white) than in the other alcoholic beverages considered (Smirnoff ice and Elephant beer). The authors attributed this difference to the higher sugar content in the less tolerated beverages [27]. The cross-sectional study conducted by Triggs et al. was based on the compilation of a questionnaire regarding dietary consumption; the aim was to evaluate the tolerance or lack thereof of the individual categories of food/beverages. As for alcoholic beverages, most subjects with CRD showed a worsening of the symptoms of the disease (>55% for beer and >50% for red wine). On the contrary, some patients who consumed beer and about 5% of red wine consumers reported good tolerance [35].

4. Studies that suggest that wine consumption plays a controversial role.

According to a subsequent prospective study by Swanson et al., red wine consumption for one week in six subjects with inactive CRD and eight subjects with inactive UC produced somewhat contrasting effects [32]. On the positive side, the parameters measured as indices of inflammation showed either no change in trends (C-reactive protein) or a decrease (stool calprotectin); in parallel, however, CRD subjects showed an increase in intestinal permeability, which should be understood as a worsening of intestinal function. The reduction in stool calprotectin was associated with the presence of resveratrol in red wine. The authors concluded that patients with inactive IBD who consume red wine on a daily basis may be at increased risk of long-term disease relapse. The study conducted by Zutshi and co-workers (2007), performed as a postal cohort study, reported that the consumption of alcoholic beverages (wine, beer, liquor) produced a worsening of symptoms in 40% of patients with CRD and no effect in 41% of them [36]. The results were independent of the type of beverage consumed.

### 3.5. Irritable Bowel Syndrome (IBS)

Irritable bowel syndrome (IBS) is the most common GI disorder in which people complain of abdominal discomfort or pain associated in some way with their bowel movements. This form can affect up to 20% of the population at least once in their life, regardless of age or sex. Abdominal pain may be accompanied by diarrhea, constipation, or both (mixed form), and abdominal bloating and distension are among the most frequently described symptoms. Some forms are transitory, while others last a lifetime. The syndrome is the result of a complex interaction between psychological, social and environmental factors. For more details, see reviews by Soares (2014) and Holtmann and co-workers (2016) [64,65]. 

Several studies have considered the role of alcohol consumption in the onset or worsening of GI symptoms in subjects with IBS. Okawa concluded that high alcohol consumption in a short period of time induces symptoms such as diarrhea, but light to moderate consumption does not lead to a significant difference in the incidence and/or severity of GI symptoms [66].

Only a very limited number of studies correlate wine consumption with the onset or worsening of gastrointestinal symptoms in subjects with IBS. However, it seems clear from the overall data collected that tolerance to wine and other alcoholic beverages depends on the subject and the amount of drink consumed. Reding and co-workers (2013) included in their study 166 women with and 48 women without IBS, with the objective of evaluating the role of daily habits in the induction of GI symptoms [39]. The results showed that moderate and light drinking determined no or weak GI symptoms, while binge drinking (mean intake: 4.9 drinks) was strictly associated with symptoms the next day.

The role of different food categories in the induction of gastrointestinal symptoms was assessed by Böhn and co-workers in 197 subjects with IBS who filled in a questionnaire [37]. The authors classified the various foods/beverages based on common characteristics. As for alcoholic beverages, both wine and beer were included among products with histamine-releasing properties and those rich in biogenic amines. Of the subjects recruited, 31% reported experiencing gastrointestinal symptoms and migraines after consuming these drinks. Cheyette and Cheyette (2016) suggested that IBS symptoms associated with the consumption of certain foods and beverages (red wine, raw fruits and vegetables) could be due to a dysregulation of the serotoninergic system [38]. This hypothesis was confirmed by a significant improvement that occurred due to the intake of a low dose of triptan, a serotonin receptor agonist. Based on these observations, the authors concluded that there may be a common pathogenesis mediated by the serotoninergic system, associated with migraines and irritable bowel syndrome. Swanson et al. (2010) reported that 43% of recruited subjects (9/21) with irritable bowel syndrome showed worsened gastrointestinal symptoms after consuming alcoholic beverages, regardless of the type or amount consumed [31]. A positive effect on constipation was observed in 8% of IBS subjects (61/766) compared to 30% of controls (60/200) [25].

On the bases of clinical data, the use of alcoholic beverages is mostly discouraged or should be reduced to moderate and occasional consumption according to the scientific associations in relation to gastrointestinal diseases.

### 3.6. Microscopic Colitis (MC)

Microscopic colitis is a chronic inflammatory disease of the intestine, the diagnosis of which requires a microscopic examination (hence the name microscopic colitis), since, based on an endoscopy, the colon appears normal or almost normal. MC mainly manifests as chronic diarrhea, without mucus or blood; it particularly affects subjects between 40 and 50 years of age, more frequently females. For further detail, see the reviews by Tome et al. (2021) and Miehlke et al. (2021) [67,68].

In the large prospective study carried out by Niccum et al. (2022), alcohol consumption was found to be proportionally associated with the risk of microscopic colitis [40]. The data obtained considering individual alcoholic beverages (light and regular beer, white and red wine, spirits) indicate that the increase in risk is greater in the case of wine consumption.

## 4. Conclusions

Based on the reported data, it is not easy to draw definitive conclusions regarding the positive or negative role of moderate wine consumption in subjects with gastrointestinal tract pathologies.

Patients report different effects of alcoholic beverages on gastrointestinal symptoms: they range from the relief of postprandial discomfort to nausea or abdominal troubles. According to Franke et al. (2004 and 2005), altered gastric emptying due to the consumption alcoholic beverages may be responsible for these symptoms [20,21].

It is quite evident that the gastrointestinal tract represents the part of the human body most influenced by the molecules (mainly alcohol) present in alcoholic beverages since, after ingestion, there is direct contact with the esophageal and gastric mucosa. The intestine can also be significantly affected by alcoholic beverages, although, in this area, the action of alcohol and other active components (such as polyphenols) is more delayed over time and is also modulated by metabolic activity.

The studies selected in this review based on the established inclusion criteria are not always numerically satisfactory, but it should be emphasized that, in some cases, the subjects included in the studies are particularly numerous (even more than 100,000), making the related conclusions more reliable. For some GI pathologies (such as IBD and IBS), the wide diffusion and the number of subjects considered in the studies allow us to draw some conclusions, even if they are based on contradictory results.

For individuals with IBD (both CRD and UC), results regarding the effects of wine consumption on gastrointestinal symptoms and quality of life are conflicting. Generally speaking, wine consumption (moderate) should be permitted only under the supervision of a medical specialist. This means that some individuals can tolerate moderate amounts of wine, although it is important that patients be informed that daily wine consumption may increase the risk of long-term disease recurrence [32].

As for the disease incidence, Casey et al. (2022) conducted a prospective cohort study (involving approximately 238,000 subjects) that did not find significant correlation with wine consumption [26].

Even in the case of subjects with IBS, a general increase in GI symptoms is observed when consuming alcoholic beverages, without any less negative behavior being observed with wine [32,37].

The effect of even moderate wine consumption on gastritis and other gastroesophageal diseases generally shows a negative trend. Stronger evidence is observed in the case of gastroesophageal reflux, which would seem to be exacerbated by the consumption of alcoholic beverages, including wine [17,18,19]. Similarly, in the only available study, wine appeared to increase the risk of microscopic colitis [40].

Summarizing as far as possible the data collected and discussed in this review, it can be concluded that:

- The consumption of alcoholic beverages (including wine) in subjects with GI diseases must be carefully considered in relation to the disease, the individual symptoms, and tolerance.
- In some cases, complete abstinence is recommended; in others, moderate and/or occasional consumption can be allowed according to medical advice.

## 5. Limitations

The general conclusions that can be drawn from what is described in this review indicate that the moderate intake of wine could be a source of discomfort in patients suffering from GI pathologies, so that medical supervision is recommended at least in the most severe forms.

The authors are aware of the limitations of this review, as the number of studies that fit our inclusion criteria is extremely low; in some cases, a single satisfactory study was identified (gastritis and microscopic colitis). The paucity of studies (and the consequent uncertainty in the conclusions) derives from several concomitant factors: (1) the difficulty in planning prospective studies that recruit large number of individuals to be followed for long periods (sometimes for years) to evaluate the association between wine consumption and gastrointestinal diseases. Even when the experimental protocol is very rigorous, it is practically impossible to eliminate biases deriving from the type of diet, smoking, physical activity, etc. (social bias); (2) the data collected are often based on retrospective surveys in which the recruited subjects describe (not always accurately) their daily habits (reporting bias); (3) the reported consumption of wine (and, in some studies, the consumption of other alcoholic beverages) could be inaccurate due to recall bias (reported via questionnaire) and a bias of prevarication (alcohol consumption is a delicate topic given the social implications and the very different positions between countries).

There are, however, other problems to be highlighted: (1) prospective studies that include the use of alcoholic beverages are not approved by the Ethics Committees of many countries; (2) studies that report in the conclusions a null or doubtful effect of moderate wine consumption on the pathology studied are not finalized in papers (poor diffusion), since the authors know that such results are rarely accepted by scientific journals as they are not considered innovative (publication bias); (3) a highly important component for the health of the intestinal tract was rarely considered in previous clinical studies: the microbiota. The microbiota is increasingly studied, and the results support its role in many pathologies (not only gastrointestinal issues), even if, in this context, the results can be contradictory and/or preliminary. Among the studies included in this review, Taladrid et al. (2022 and 2023) compared the taxonomic classification of the microbiota between UC patients (*n* = 10) and healthy subjects (*n* = 8); they confirmed the presence of intestinal dysbiosis in the first group [33,34]. Some recent studies can deepen our understanding of this topic [69,70].

It follows that what is described in this review presents important limitations that can be overcome only by promoting new studies on humans. The International Organisation of Vine and Wine (OIV), in which most of the authors of this review participate, includes among its activities the collection, evaluation, discussion, and dissemination of knowledge and the promotion of research on the role of the consumption of grapes and grape derivatives in the general well-being of consumers. As in this review, it also promotes reviews focused on specific pathologies to orient readers and the scientific world in this context. The authors hope that this review will provide useful information for the planning of new experimental studies that can confirm or deny what is known today.

## Figures and Tables

**Table 1:** Summary of the studies selected according to the inclusion criteria.

Disease	Study Details	Number of Subjects	Beverages Included and Method	Objectives of the Study	Main Outcomes	Ref.
Gastritis and Gastroesophageal Disease (GERD)	Epidemiological study: population-based case-control study	9444 subjects (age: 50–74 yrs)	Beer and wine	To evaluate the association of alcohol consumption with chronic atrophic gastritis (CAG) among older adults. Serological levels of pepsinogen I and II were measured as diagnostic parameters of the disease and antibodies vs. *Helicobacter pylori* as an index of infection.	Moderate alcohol consumption (<60 g/week) was associated with a significant reduction in CAG risk (odds ratio 0.71) when compared to abstainers. Effects were observed with both beer and wine.	[15]
	Clinical trial: cross-over intervention study	14 healthy male volunteers (mean age 25 yrs; range 18–35). Not consumers or moderate consumers of alcoholic beverages (less than 1–3 g alcohol/day). The final evaluation was performed on 13 subjects.	360 mL of red wine (13% alcohol) or tap water.	To monitor oesophageal motility and esophagogastric pH in an ambulatory 24-h study. The participants received the test beverage with a meal. Each subject was their own control. Measures were taken with a portable recording system during the meal (30 min), postprandial (3 h), and after 8 h supine.	The only esophageal motility change was an increase in the number of high-amplitude waves during wine consumption (1.6 vs. 1.2 of water, *p* = 0.02). The percent reflux time increased during the postprandial period after wine ingestion in comparison with water. Red wine induced heartburn in 2 out of 13 healthy subjects. No significant changes in gastric pH after wine ingestion compared with water during either the postprandial or supine period.	[16]
	Clinical trial: cross-over intervention study	20 healthy volunteers (13 M; 7 F; age: 23–37 years)	300 mL of white wine (8% alcohol) or 8% alcohol solution or tap water with a standardized meal in random order.	To verify whether white wine modified oesophageal peristalsis and acid clearance. Acid clearance was measured via the instillation of 15 mL 0.1 N HCl into the distal oesophagus.	White wine temporarily alters oesophageal clearance by disrupting the initiation of secondary peristalsis and by increasing ineffective contractions. Ethanol alone (8% alcohol solution) does not produce the same effects as white wine.	[17]
	Clinical trial: cross-over intervention study	Twelve healthy volunteers (5 M; 7 F; median age: 31 yrs, range 25–37). They did not usually drink alcoholic beverages.	300 mL of white wine or tap water.	To explore the pathogenesis of prolonged reflux duration. The participants received wine or water randomly with a standardized meal. Oesophageal pH and motility were evaluated using a glass pH electrode and a strain gauge manometry probe.	Reflux events of long duration were associated with the intake of white wine.	[18]
	Clinical trial: cross-over intervention study	25 participants (18 M; 7 F; mean age: 54 yrs, range 24–84). Clinical evidence: 15 had reflux oesophagitis, 10 non-erosive reflux.	300 mL of white wine (17 subjects), or 500 mL beer (8 subjects) or water.	To measure the effect of beverages on postprandial reflux. The participants received the test beverage randomly with a standardized meal; after 2 days, they received an equivalent volume of tap water. The oesophageal pH was measured using a glass electrode.	An increase in reflux was observed with both wine and beer in both groups of patients.	[19]
Gastrointestinal motility	Clinical trial: randomized cross-over intervention study	10 healthy participants	500 mL of beer, red wine or corresponding alcoholic solutions (4 and 10%, respectively), 500 mL of 5.5 or 11.4% glucose solution and water. Moreover, 125 mL of whisky or 40% alcohol solution was followed by 125 mL of water.	To compare the effect of alcoholic beverages on gastric emptying using ultrasonography of the antrum. The fasted participants received randomly, on separate days, the test beverage via oral gavage.	Inhibition of gastric emptying was observed with the 4%, 10%, and 40% (*v*/*v*) alcohol solutions. The inhibitory effect of beer and red wine, but not of whisky, was stronger than that of their corresponding ethanol solutions. Mean half-emptying times were 72.6 min for red wine, 39.2 min for beer, and 26.4 min for whisky.	[20]
	Clinical trial: randomized cross-over intervention study	16 healthy males (mean age ± SD 29 ± 2.1 yrs). They were non-smokers and were not regular consumers of alcoholic beverages	300 mL of 4 or 10% ethanol, beer, red wine, water, 5.5 or 11.4% glucose.	To assess the effect of alcoholic beverages on gastric emptying. The participants randomly received the test beverage once weekly with a low-calorie (270 kcal) or high-calorie (740 kcal) solid meal. Gastric empty was measured via ultrasonography.	4 and 10% ethanol solutions and alcoholic beverages (beer and red wine) caused a prolongation of gastric emptying after a solid meal. The inhibitory effect of red wine exceeds that of the corresponding alcoholic solution. The inhibitory effect was independent from the caloric content of the meal.	[21]
	Clinical trial: cross-over intervention study	A 65-year-old woman with Dumping syndrome characterized by GI symptoms after meals (abdominal pain, bloating, nausea, vomiting and diarrhea).	8 ounces (240 mL) of Bogle Merlot wine (14.5°)	To study 4-h gastric emptying expressed as the percentage of isotope emptied, after a standardized isotope-labelled meal (255 kcal).	Gastric emptying in this subject at baseline was too fast and was normalized when wine was associated with the meal. Values of emptying at 120 min were 55% vs. 89% at baseline (normal value <80%). The intake of wine eliminated the post-prandial symptoms.	[22]
	Clinical trial: randomized cross-over intervention study	23 healthy volunteers (21–32 yrs; 33.3% M; 66.7% F), negative at *H. pylori* infection; no systematic use of alcoholic beverages. One volunteer participated to 2 sessions and six to 3 sessions.	Participants randomly received 400 mL beer (4.7 %vol ethanol) or 200 mL red wine (13.7 %vol) or 100 mL whisky (43.5 %vol) or corresponding volumes of fluids with equivalent alcoholic content with a solid meal (1485 kJ/355 kcal).	To evaluate gastric myoelectrical activity and emptying, orocecal transit time and gallbladder emptying were monitored noninvasively by electrogastrogram and ultrasonography of gallbladder.	After a solid meal, all alcoholic beverages inhibited gastric and gallbladder emptying. The magnitude of the effect was correlated with the alcoholic content. As regards the orocecal transit, different effects are observed depending on the type of beverage: whiskey causes a delay, while beer and red wine do not.	[23]
	Clinical trial: randomized cross-over intervention study	10 healthy male volunteers with no history of GIDs (mean age ± SD 30.8 ± 7.6; range 21–43).	Wine meal: 450 g red wine (alcohol 9.5 g/dL) in 900 g of total meal (882 kcal, 545 from wine). Low-alcohol wine meal: as above, with the previously mentioned wine boiled for 7 min (alcohol 1.13 g/dL).	To assess the effect of an ethanol-containing meal (wine) on gastric emptying using a dual radioisotopic method. The participants received meals in a random order. Seven subjects also received wine and low-alcohol wine without a meal: five received the meal free from wine.	No differences in gastric emptying were observed between the administration of a meal without or with wine/low-alcohol wine. No difference was evident when the beverages were administered without a meal.	[24]
	Epidemiological study: population-based case-control study	200 healthy controls (84 M; 116 F; mean age ± SD 50.2 ± 1.2 yrs); 122 patients with chronic constipation (16 M; 106 F; mean age ± SD 51.9 ± 1.3 yrs) 766 patients with IBS with constipation 199 M; 567 F; mean age ± SD 51.0 ± 0.5 yrs)	Different food and beverages considered: beer, black tea, coffee and wine.	To evaluate via a questionnaire the effect of foods and beverages on stool consistency in healthy and chronically constipated populations	In all groups, black tea was classified as a constipating agent, while coffee, beer, and wine were stool softeners. The promoting effect of wine was present in 21% of constipated patients, 8% of IBS–constipated patients, and 30% of controls.	[25]
Inflammatory Bowel Disease (IBD) and Chron’s Disease (CRD) and Ulcerative Colitis (UC)	Epidemiological study: prospective cohort study	237,835 participants from the Nurses’ Health Study, Nurses’ Health Study II, and Health Professional Follow-Up Study. Mean age at baseline: 46.4 yrs (26–80 yrs)	Beer, wine, liquor.	To evaluate the correlation between alcoholic beverage consumption and the risk of CRD and UC via a questionnaire filled out every four years.	Moderate consumption of beer (>1–4 servings/week) was associated with a lower risk of CRD (0.50). Higher risk of UC was observed for the intake of >4 servings/week of liquor. No significant association was found between wine intake and the risk of CRD or UC.	[26]
	Clinical trial: cross-over intervention study	12 healthy subjects (5 M; 7 F; age 21–52); 20 CRD in remission (9 M; 11 F; age 18–50).	Red wine, white wine, Smirnoff ice, Elephant beer, pure ethanol. For all beverages, the intake was based on: 36 g alcohol for men and 24 g alcohol for women.	To evaluate the effect of alcoholic beverages (randomly consumed) on abdominal discomfort in CRD. After 48 hrs of an alcohol-free diet and at 2-week intervals, participants randomly received one of the 4 test beverages or ethanol solution. Serum ethanol and plasma glucose concentrations were measured at 0, 30, 60, 90, 120, and 180 min. A self-reported pain symptom score was used.	No difference in alcohol absorption was detected between CRD patients and controls. No changes in the acute-phase inflammatory markers were found during the study period. When compared to controls, all drinks determined an increase in abdominal pain in CRD patients. Compared to the other beverages tested, red and white wines determined (1) a lower plasma sugar concentration and (2) a lower effect on self-reported abdominal pain in CRD patients.	[27]
	Epidemiological study: prospective cohort study	Participants: IBD (mean age ± SD at start 70.2 ± 8.0 yrs; 49.1% M; 50.9% F) 2027 with CRD 4334 with UC 734 with diagnosis for both diseases Non-IBD 495,410 (mean age ± SD at start 69.5 ± 8.1 yrs; 45.5% M; 54.5% F).	Red wine, champagne and white wine, beer, cider, spirits and fortified wine.	To evaluate the correlation between intake of alcoholic beverages and risk of IBD. Participants responded to: “About how often do you drink alcohol?”. The frequency was classified into: (1) high frequency (≥3 times/week); (2) low frequency (<3 times/week); and (3) never/special occasions only.	Compared with abstainers, red wine consumers showed a lower risk of IBD (23% with 3–4 glasses/week). High frequency and high doses of white wine and champagne, low frequency and high doses of spirits, and high doses of beer and cider appeared to increase the risk.	[28]
	Epidemiological study: prospective cohort study	81 UC patients 43 M (mean age 53 yrs; 26–78 yrs) 38 F (mean age 47 yrs; 19–74 yrs).	Beer, wines (red, white, and sweet) and spirits	A validated 7-day diet diary was filled. Clinical data were collected, and patients were examined using rigid or flexi-sigmoidoscopy and graded (score 0–6). Scores were confirmed via histological examination.	Beer and wines were responsible for increased UC activity. No correlation was found with spirits. Sulphites more than alcohol could play a role in disease process.	[29]
	Epidemiological study: population-based case-control study	167 UC patients diagnosed in Uppsala country from 1945 to 1964 (87 M; 80 F; at interview mean age 13 yrs; range 4–35 yrs). 167 sex-matched controls.	Beer, wine, liquor.	To study, via a face-to-face interview, the role of socioeconomic, dietary (including alcoholic beverages), and personal habits on UC incidence.	No difference was found in the correlation between the consumption of alcoholic beverages and the incidence of UC when the IBD group was compared to the control population.	[30]
	Epidemiological study: cross-sectional study	Participants: 52 with inactive CRD (20 M; 32 F; mean age 42.4 yrs) 38 with inactive UC (14 M; 24 F; mean age 38.5 yrs).	Beer, wine, and liquor	To evaluate, using validated questionnaires, the correlation between alcoholic beverage consumption and disease activity (the Crohn’s disease activity index or ulcerative colitis clinical activity index).	Patients with inactive IBD consumed alcoholic beverages similarly to the general population. Patients consuming alcoholic beverages described a worsening of GI symptoms (75% in CRD and UC patients), but no correlation was observed between the quantity/type of alcoholic beverage and the severity of GI symptoms.	[31]
	Epidemiological study: prospective cohort study	Participants: 6 with inactive CRD (4 M; 2 F; median age 31 yrs) 8 with inactive UC (4 M; 4 F; median age 45 yrs) 7 controls (3 M; 4 F; median age 24 yrs).	Red wine (1–3 glasses/day for a week).	To evaluate the role of moderate red wine consumption on IBD. A validated questionnaire was used to confirm the inactive status of disease (Crohn’s disease activity index or ulcerative colitis clinical activity index). Other parameters considered: C-reactive protein and stool calprotectin (inflammatory indices); intestinal permeability.	One week of moderate wine consumption determined no significant change in the clinical disease activity score or C-reactive protein in IBD subjects. Compared to controls, the researchers observed: (1) a significant decrease in stool calprotectin from the starting values; (2) a significant increase in intestinal permeability.	[32]
	Clinical study: case–control intervention study	10 UC patients in active phase (both sexes; 18–42 yrs) 8 healthy subjects for the study of the microbiome.	Red wine (125 mL × 2 doses/day for 4 weeks)	To assess the role of moderate red wine consumption on clinical parameters and severity of symptoms. After 2 weeks of wash out (no wine, low-polyphenol diet-LPD), 5 UC patients consumed red wine, and 5 UC patients did not. Intestinal symptoms were collected using a validated questionnaire. Serum and urine were collected to measure biochemical parameters. Intestinal dysbiosis was also considered.	Regular and moderate red wine intake improved the clinical situation and the GI symptoms of patients in the active phase.	[33]
	Clinical study: case–control intervention study	10 UC patients in the active phase (both sexes; 18–42 yrs) 8 healthy subjects for the study of the microbiome.	Red wine (125 mL × 2 doses/day for 4 weeks)	To evaluate the effects of moderate red wine consumption on the clinical status and symptoms in UC subjects. After 2 weeks of wash out (no wine, low-polyphenol diet-LPD), 5 UC patients consumed red wine, while 5 UC patients did not. Intestinal symptoms were collected via a validated questionnaire. Serum and urine were collected and analyzed (glucose, lipids, hepatic enzymes, etc.) using an automated biochemical auto-analyzer.	Moderate red wine intake improved the quality of life of UC patients, at least partially mediated by a significant improvement in serum iron and transferrin saturation index (biomarkers of anaemia) and a reduction in the severity of active intestinal symptoms. Calprotectin levels (faecal marker of UC) decreased in intervention group. An effect on the oral and intestinal microbiome was also found in the intervention group, in term of stabilization of biodiversity and increase of positive microbiota.	[34]
	Epidemiological study: cross-sectional study	446 CRD subjects (136 M; 283 F) Age at diagnosis: 9.3% <17; 65.8% 17–40; 24.9% >40.	Beer and red wine	To evaluate the self-reported dietary tolerance and intolerance to specific food/beverages using a dietary questionnaire.	Many subjects avoided alcohol based on medical advice, not because of personal negative experience. Beer worsened symptoms in > 55% of patients but helped a few. Red wine worsened symptoms in >50% of patients but was well tolerated by approximately 5%.	[35]
	Epidemiological study: postal cohort study	1220 patients with CRD from the Cleveland Clinic Digestive Disease Centre Database	Wine, beer, liquors, or mixed alcoholic beverages	To evaluate the role of dietetic factors on clinical symptoms	A worsening of symptoms was observed in 40% of patients consuming alcoholic beverages, while no change was reported by 41% of patients in the same group. No difference was observed among the alcoholic beverages included.	[36]
Irritable Bowel Syndrome (IBS)	Epidemiological study: cross-sectional study	197 IBS participants (55 M; 142 F; mean age 35 yrs; range 18–72)	Beer and wine	The correlation between different food items and quality of life in IBS was evaluated using a non-formally validated questionnaire	31% of subjects experienced GI symptoms. No difference between beverages was reported.	[37]
	Case report	A 50-year-old Caucasian male characterized by migraine and acute GI distress triggered by common IBS trigger foods such as insoluble fibre, red wine and large/rich meals.	Wine	To evaluate the putative contributions of wine to the observed symptoms resulting from the dysregulation of the serotoninergic system.	The hypothesis was confirmed by the highly satisfactory improvements in the described symptoms obtained with a low dose of triptan (a seretonin receptor agonist).	[38]
	Epidemiological study: population-based case-control study	766 IBS patients with constipation (199 M; 576 F; mean age ± SD 51.0 ± 0.5 yrs) 200 healthy controls (84 M; 116 F; mean age ± SD 50.2 ± 1.2 yrs)	Different food and beverages considered: the beverages included beer, black tea, coffee and wine.	To evaluate via a questionnaire the effect of foods and beverages on constipation in healthy and IBS–constipated populations.	In both groups, black tea was classified as constipating, while coffee, beer, and wine were stool softeners. The effect of wine was present in 8% of IBS patients and 30% of controls.	[25]
	Epidemiological study: prospective observational study	Women with (166) or without (48) IBS (mean age 32 yrs; 18–48 yrs) with a similar pattern of alcohol intake	Beer, wine and liquor; 1 drink = 118 mL wine 237 g of beer 1 shot of liquor.	To evaluate the role of habits in the presence of GI symptoms by using a daily diary reporting the number of drinks of alcoholic and caffeine-containing beverages consumed, cigarette smoked and level of stress.	The following symptoms were considered: abdominal pain and bloating, intestinal gas, nausea, stomach pain, heartburn, indigestion, diarrhea, and constipation. Moderate and light drinking determined no or weak GI symptoms, while binge drinking (mean intake: 4.9 drinks) was strictly associated with symptoms the next day. No detail is reported on the possible difference between the beverages used.	[39]
	Epidemiological study: cross-sectional study	39 participants (7 M, 32 F; mean age 53.2 yrs)	Beer, wine and spirits	To evaluate, by using validated questionnaires, the correlation between alcoholic beverages consumption and worsening of GI symptoms.	Of the current drinkers (21/39), 43% of patients described a worsening of GI symptoms when alcohol was consumed. No correlation was observed between the quantity or type of alcoholic beverage consumed and severity of GI symptoms.	[31]
Microscopic colitis (MC)	Epidemiological study: prospective cohort study	209,902 female participants from the Nurses’ Health Study and Nurses’ Health Study II (mean age at baseline 45.5 yrs, range 28.5–66.7)	Light and regular beer, white and red wine, liquor	To evaluate the association between alcohol intake and the adjusted hazard risk (aHR) of microscopic colitis. Alcohol consumption was obtained using a food frequency questionnaire (updated every 4 years). MC was diagnosed according to histopathological data.	Higher alcohol consumption was associated with an increased risk of MC. When stratified by beverage type, the aHR according to every 2 servings/week seemed strongest with wine (1.08) as compared to beer (1.01) or liquor (1.00).	[40]

M = male; F = female; yrs = years; hrs = hours; GID = Gastrointestinal disease; CRD = Crohn’s Disease; UC = ulcerative colitis.

## Abbreviations

The following abbreviations are used in this manuscript:

GI	Gastrointestinal
IBD	Inflammatory bowel disease
IBS	Irritable bowel syndrome
GERD	Gastroesophageal disease
GID	Gastrointestinal disorder
CRD	Chron’s disease
UC	Ulcerative colitis
MC	Microscopic colitis
CD	Celiac disease
CAG	Chronic atrophic gastritis
